# Preparation of a Ni-P-nanoPTFE Composite Coating on the Surface of GCr15 Steel for Spinning Rings via a Defoamer and Transition Layer and Its Wear and Corrosion Resistance

**DOI:** 10.3390/ma16124427

**Published:** 2023-06-16

**Authors:** Shunqi Mei, Cong Zhou, Zekui Hu, Zhi Xiao, Quan Zheng, Xuhui Chai

**Affiliations:** 1 Hubei Digital Textile Equipment Key Laboratory, Wuhan Textile University, Wuhan 430073, China; sqmei@wtu.edu.cn (S.M.);; 2 School of Mechanical & Electrical Engineering, Zhongyuan University of Technology, Zhengzhou 450007, China

**Keywords:** electroless plating, composite coating, wear resistance, corrosion resistance, GCr15 steel

## Abstract

In this study, a method of preparing a Ni-P-nanoPTFE composite coating on the surface of GCr15 steel for spinning rings is proposed. The method incorporates a defoamer into the plating solution to inhibit the agglomeration of nano-PTFE particles and pre-deposits a Ni-P transition layer to reduce the possibility of leakage coating. Meanwhile, the effect of varying the PTFE emulsion content in the bath on the micromorphology, hardness, deposition rate, crystal structure, and PTFE content of the composite coatings was investigated. The wear and corrosion resistances of the GCr15 substrate, Ni-P coating, and Ni-P-nanoPTFE composite coating are compared. The results show that the composite coating prepared at a PTFE emulsion concentration of 8 mL/L has the highest concentration of PTFE particles (up to 2.16 wt%). Additionally, its wear resistance and corrosion resistance are improved compared with Ni-P coating. The friction and wear study shows that the nano-PTFE particles with low dynamic friction coefficient are mixed in the grinding chip, which gives the composite coating self-lubricating characteristics, and the friction coefficient decreases to 0.3 compared with 0.4 of Ni-P coating. The corrosion study shows that the corrosion potential of the composite coating has increased by 7.6% compared with that of the Ni-P coating, which shifts from −456 mV to a more positive value of −421 mV. The corrosion current reduces from 6.71 μA to 1.54 μA, which is a 77% reduction. Meanwhile, the impedance increased from 5504 Ω·cm^2^ to 36,440 Ω·cm^2^, which is an increase of 562%.

## 1. Introduction

GCr15 steel is widely used in the automotive, aerospace, textile, and other industrial fields, mainly to manufacture rotary motion core components, such as bearings, balls, spinning rings, etc. [1,2,3]. It provides a low and stable friction coefficient, though it can easily wear and rust, thus, usually requiring surface treatment. For example, in the ring spinning process, the steel ring provides a jumping and sliding track for the traveler, with a running speed of up to 30 m/s, and due to the high humidity of the workshop environment, it is prone to premature wear and corrosion [4]. At the same time, it is necessary to shorten the running-in period of the steel ring and the traveler, and shorten the formation time of the fiber lubricating film, which is the key for maintaining a low friction coefficient.

At present, the commonly used surface treatment technologies for GCr15 steel include thermal spraying, thermal diffusion, vapor deposition, electroless plating, electroplating, and laser texturing, among others [5,6,7]. Fang et al. [8] thermally sprayed a Ni60A coating on the surface of GCr15 steel and carried out an induction remelting treatment; the hardness of the coating was as high as 818 HV. Tian et al. [9] used vapor phase chromizing technology to obtain a chromizing layer on the surface of GCr15 steel, and the hardness of the chromizing layer reached 1600 HV. Xie et al. [10] coated titanium on the surface of a Φ 8 mm GCr15 steel ball and performed ball milling treatment to obtain a titanium-hardened layer with a hardness of 895 HV on the surface of the steel ball. Li et al. [11] carried out laser texturing on the surface of GCr15 steel and studied its friction performance under the lubrication of a suspension of graphene and liquid crystal 4-n-pentyl-4-cyano biphenyl (5CB). The results showed that the texture can store lubricating oil and effectively reduce the friction of the contact area. On the one hand, the above process can significantly increase the hardness of the GCr15 steel surface and improve the wear resistance to a certain extent; however, the high hardness will prolong the running-in period of the steel ring and the traveler, as well as the formation time of the lubricating film, and the process is complicated and costly. On the other hand, the laser surface texture can form pits on the surface of the steel to store lubricating oil, but it is unsuitable for the spinning industry. The pits affect the dimensional accuracy, and the lubricating oil contaminates the yarn. In summary, the surface treatment processes described above are not appropriate for spinning rings.

Electroless nickel plating (ENP) has the advantages of being a simple process, easy to operate, and environmentally friendly compared with electroplating. The prepared nickel–phosphorus coating exhibits good resistance to wear and corrosion. Simultaneously, it can further introduce various functional particles, such as SiC, Al_2_O_3_, PTFE, MoS_2_, and others, to co-deposit with the nickel–phosphorus alloy to form a composite coating, endowing the coating with special properties, such as super hardness and self-lubrication [12,13,14,15]. Among them are soft nano-PTFE particles, which have a considerably low dynamic friction coefficient [16,17,18]. Additionally, the Ni-P-nanoPTFE composite coating, deposited after being incorporated into the coating, has self-lubricating properties. Many researchers have successfully prepared Ni-P-nanoPTFE composite coatings on metals and nonmetals and studied the effects of plating solution components, process parameters, dispersion methods, and PTFE content on the wear and corrosion resistance of the coatings [19,20,21]. The recent research mainly focuses on the performance of combining Ni-P-nanoPTFE composite coatings with other coatings [22] or how to increase the PTFE particle content in Ni-P-nanoPTFE composite coatings on substrates in a short time [23]; however, the research on how to ensure the uniform dispersion of nano-PTFE particles in Ni-P-nanoPTFE composite coatings and reduce the occurrence of missing plating is not perfect. Relevant studies have shown that the combination of an appropriate amount of cationic surfactants and fluorocarbon nonionic surfactants can significantly improve the dispersion of nano-PTFE particles in the solution [24,25,26], but this will increase the foaming properties of the plating solution. The formation of a large number of foams causes the agglomeration of nano-PTFE particles to float, reducing the total amount of PTFE in the plating solution and the PTFE content in the final composite coating. Experiments revealed that the introduction of defoamers not only offsets the foaming properties of surfactants but also causes the bubbles in the bath to escape faster. At the same time, to further improve the dispersion of PTFE particles, the composite plating deposition process must be supplemented with mechanical stirring and ultrasonic stirring, whereas nano-PTFE particles generally do not participate in the plating solution reaction and rely solely on weak physical adsorption to remain on the surface of the substrate during deposition. Additionally, it is finally wrapped in a nickel–phosphorus alloy and dispersed into the coating; however, because the flow rate of the plating solution at the edge of the surface of the substrate to be plated is higher than that in the center, it is difficult for PTFE particles to stay at the edge position. This phenomenon will cause the edge of the part to miss plating, which seriously affects the coating quality. This can often be improved by precoating nickel or Ni-P coating [27,28,29,30].

This paper proposes the use of a Ni-P transition layer to improve the leakage plating phenomenon of a Ni-P-nanoPTFE composite coating, followed by the introduction of a defoaming agent to offset the side effects of surfactant foaming and PTFE agglomeration when preparing the Ni-P-nanoPTFE composite coating. The deposition principle of the composite coating by using a transition layer and introducing a defoamer was investigated, and the influence of different PTFE emulsion concentrations on the coating was explored. A performance comparison study was conducted on the wear and corrosion resistance of the GCr15 steel substrate, Ni-P coating, and Ni-P-nanoPTFE composite coating.

## 2. Materials and Methods

### 2.1. Materials

An annealed GCr15 steel plate was used as the experimental substrate, with a hardness of approx. 180 HV. The detailed chemical composition is shown in Table 1. Before the experiment started, it was first cut using a wire cutting machine into a standard block with a size of 20 mm × 15 mm × 5 mm, and a hole was punched at one end. Then, four specifications (160#, 320#, 600#, 1000#) of silicon carbide sandpaper were then used to sand and polished it to a mirror-like surface, followed by washing and drying.

### 2.2. Alkaline Cleaning and Degreasing and Activation

Before electroless plating, the substrate must undergo alkali cleaning, degreasing, and activation. First, a nylon rope was passed through the small hole of the standard block to fasten the sample, followed by soaking in a preconfigured 60 °C alkali washing solution (20 g/L, NaOH; 30 g/L, Na_2_CO_3_) for 5–10 min to remove the surface grease. After removing it, it was cleaned with deionized water and placed into an ultrasonic cleaner for 10 min with absolute alcohol as the solvent. Finally, it was immersed in 10% hydrochloric acid to activate it for about 30 s to remove the surface oxide layer. When the surface of the sample turned grey and small bubbles escaped, it was immediately placed in the prepared chemical plating solution for deposition.

### 2.3. Electroless Deposition of Ni-P/Ni-P-nanoPTFE Coating

The plating solution composition and process parameters of the electroless Ni-P/Ni-P-nanoPTFE coating are shown in Table 2, which is based on my previous experimental research. Nano-polytetrafluoroethylene was made from PTFE emulsion produced by Daikin Corporation of Japan, with a solid content of 60 wt%, a particle size of approx. 200 nm, and a density of 1.5 g/cm^3^. The cationic active agent cetyltrimethylammonium bromide (CTAB) was selected as the surfactant, the non-ionic active agent was FC-4430 produced by 3M Company (Maplewood, MN, USA), and the industrial defoamer KR-XP96 was selected as the defoamer.

**Figure 1:**
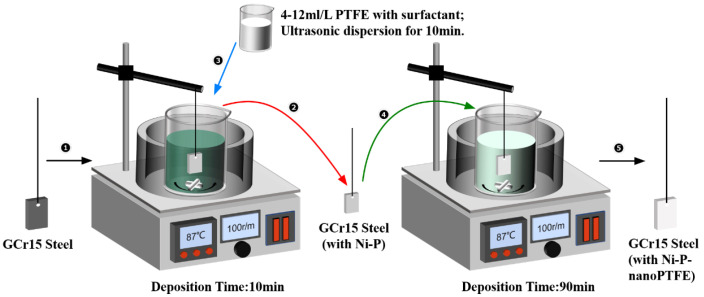
Schematic diagram of the Ni-P-nanoPTFE coating preparation process.

The schematic diagram of the Ni-P-nanoPTFE composite electroless plating experiment is shown in Figure 1. (1) According to the drug content in Table 2, take an appropriate amount of NiSO_4_·6H_2_O, NaH_2_PO_2_·H_2_O, C_6_H_8_O_7_·H_2_O, NaC_2_H_3_O_2_·3H_2_O, H_2_NCSNH_2_ drugs. They are mixed and dissolved in a small amount of deionized water, and mixed and fixed to 1 L. Then, heat it in a water bath to 85 °C to keep it warm, adjust the PH to 4.4 with 10% H_2_SO_4_ and NH_3_·H_2_O, and configure the Ni-P plating solution. (2) Deposit the matrix after the pretreatment process in the Ni-P plating solution for 10 min to obtain the Ni-P transition layer. At the same time, according to the drug content in Table 2, take an appropriate amount of PTFE emulsion, surfactant and defoamer, mix it to 50 mL and disperse it ultrasonically for 10 min, and configure the additional solution. (3) Remove the substrate from the Ni-P plating solution and place in deionized water at 80 °C to keep warm. Then, mix the dosing solution with Ni-P plating solution and mix at 100 rpm for 10 min to prepare Ni-P-nanoPTFE composite plating solution. Then, deposit the matrix coated with the Ni-P transition layer in the composite plating solution for 90 min to obtain Ni-P/Ni-P-nanoPTFE combination coating. (4) Directly place the matrix into the Ni-P plating solution and deposit for 1 h to obtain the Ni-P coating sample in the wear and corrosion resistance comparison experiment.

### 2.4. Coating Characterization

The micromorphology and comprehensive properties of Ni-P-nanoPTFE composite coatings were characterized as follows: (1) The coating surface and cross-sectional morphology were observed by OLYMPUS-DSX-HRUF optical microscope and Oxford scanning electron microscope, the coating thickness (μm) was measured and the deposition rate (μm/h) was calculated; each sample was measured three times for average. (2) The microhardness of the coating is measured by an HV-1000 hardness measuring instrument, the set load is 100 g, the holding time is 10 s, and each sample is averaged by measuring 5 points. (3) The coating structure is detected by Empyrean X-ray diffractometer, using Cu target Ka radiation, accelerating voltage is 40 KV, the working current is 40 mA, X-ray incidence wavelength L = 0.1542 nm, the scanning speed is 6°/min, the step size is 0.02°/s, and angle (2θ) is 20~80°. (4) The chemical composition of the coating is measured by the energy dispersion spectrometer that comes with the above scanning electron microscope, and the test elements are Ni, P, and F. (5) The friction and wear performance is measured by UMT-3 reciprocating friction testing machine, the friction pair is selected using a GCr15 steel ball with a diameter of 10 mm, and the microhardness is 810 HV. The load is set to 10 N, the reciprocating frequency is 2 HZ, and the friction stroke is 5 mm. (6) The corrosion resistance is measured by CH1660E electrochemical workstation, the classic three-electrode system is selected, the reference electrode (RE) is the saturated calomel electrode, the auxiliary electrode (CE) is the platinum electrode, the research electrode (WE) is the sample to be tested, the corrosion medium selected is a 3.5% NaCl solution, the initial electrode is set to −0.7 V, the end potential is −0.2 V, the scanning speed is 5 mV/s, and it is carried out at room temperature.

## 3. Results

### 3.1. Deposition Process of Ni-P-nanoPTFE with a Defoamer and Transition Layer

**Figure 2:**
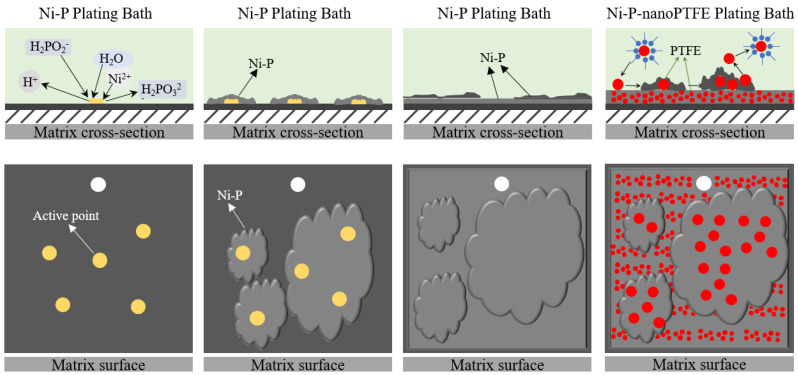
Deposition schematic of Ni-P-nanoPTFE composite coating. The yellow circle represents the active point on the surface of the substrate, while the red represents the PTFE particles embedded in the coating.

Figure 2 shows the deposition process of Ni-P-nanoPTFE composite coating with hypophosphite as a reducing agent under acidic conditions.

First, there are catalytic active sites on the surface of the GCr15 steel substrate after pretreatment. According to the mechanism of atomic hydrogen, hypophosphite reacts with water to generate atomic hydrogen at the active site, a part of atomic hydrogen combines with nickel ions and phosphorus ions to reduce it to nickel-phosphorus alloy, and a small amount of atomic hydrogen combines to form hydrogen gas as a by-product that escapes. The reaction equation is as follows (‘ad’ indicates that atomic hydrogen is adsorbed on the action potential of the substrate):

(1) $$H_{2}{\mathrm{PO}}_{2}^{-}+H_{2}O\to {\mathrm{HPO}}_{3}^{2-}+H^{+}+2H_{\mathrm{ad}}$$

(2) $${\mathrm{Ni}}^{2+}+2H_{\mathrm{ad}}\to \mathrm{Ni}+2H^{+}$$

(3) $$H_{2}{\mathrm{PO}}_{2}^{-}+H^{+}+H_{\mathrm{ad}}\to 2H_{2}O+P$$

(4) $$2H_{\mathrm{ad}}\to H_{2}$$

Secondly, there are a large number of active sites on the surface of the substrate, and the nickel–phosphorus alloy is simultaneously reduced on multiple active sites. In the very thin transition area between the substrate and the plating solution contact surface, the nickel–phosphorus alloy is continuously connected at multiple points along the surface of sheets and finally covers the entire substrate. The nickel–phosphorus alloy’s deposition method is layered accumulation growth. The water-insoluble nano-PTFE particles are physically suspended in the plating solution after the surface tension is reduced by the action of the surfactant, and the nano-PTFE particles weakly adsorb and stay on the surface of the substrate with slow stirring. One part is wrapped by the nickel–phosphorus coating produced along the substrate, and the other part returns to the plating solution due to stirring, and finally, the Ni-P-nanoPTFE composite coating is formed by continuous layer-by-layer stacking.

**Figure 3:**
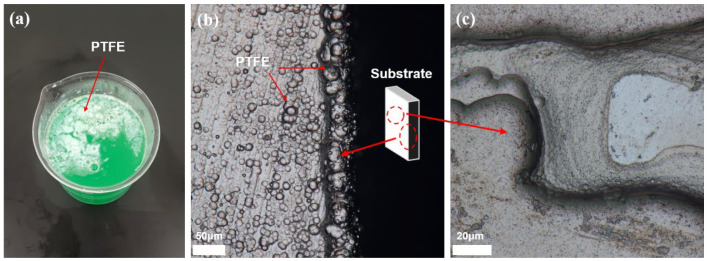
Experimental diagram of Ni-P-nanoPTFE prepared without introducing a defoamer and Ni-P transition layer. (**a**) shows a physical schematic of the plating solution without defoamer, and (**b**,**c**) are micromorphological diagrams of the edge and surface of the coating obtained after the matrix is deposited in the plating bath without defoamer.

Figure 3 shows a physical picture of the plating solution of the Ni-P-nanoPTFE coating prepared by not using a defoamer and Ni-P transition layer, as well as the microscopic topography of the coating surface.

It can be seen from Figure 3a that the color of the plating solution is opaque green, with some white polymers floating on top, while the original Ni-P solution is clear and transparent green. The cause of the above phenomenon is that a part of the white nano-PTFE particles is evenly dispersed in the plating solution, forming a colloid to make the plating solution opaque, while the other part of the white nano-PTFE particles is agglomerated into polymers and floats on the surface of the plating solution. This situation is unfavorable for the deposition of the Ni-P-nanoPTFE coating on the surface of the substrate; not only will the total amount of PTFE particles in the original plating solution be reduced, but the formed agglomerates will also be easily coated on the surface of the substrate, hindering the deposition of the composite coating. PTFE particles easily agglomerate. The introduction of surfactants and the continuous escape of hydrogen, a by-product of electroless plating, will lead to a large amount of foam and aggravate the agglomeration of PTFE particles. An appropriate amount of defoamer can inhibit the generation of foam. Figure 3b,c are failure examples of two Ni-P-nanoPTFE coating depositions. Figure 3b shows the junction of the edges and corners of the base standard block. It can be seen that the morphology of the coating at the boundary is very uneven, with many spherical and nodular protrusions; the horizontal coating and the vertical coating can also be seen clearly. This indicates that the nano-scale PTFE particles in the plating solution are slightly agglomerated, and the agglomerates are co-deposited with the nickel–phosphorus alloy. Such coatings exhibit inconsistent performance and poor bonding. Figure 3c shows that the standard block sample has missing plating. The Ni-P coating has a layer-by-layer growth mode. Since the nano-PTFE particles do not participate in the reaction in the electroless plating, they are only adsorbed on the surface of the substrate through weak physical action, which harms the deposition of the coating. At the same time, nano-PTFE particles are insoluble in water and require physical stirring to remain dispersed in the plating solution. The interaction of the two effects makes depositing Ni-P-nanoPTFE composite coatings on the edge of the matrix standard block difficult. If the surface of the substrate is not uniformly plated with a layer of nickel–phosphorus alloy at the beginning, it will affect the second layer and the subsequent multilayers, and the layer-by-layer stacking will cause missing plating on the macroscopic level; this can be improved by the Ni-P transition layer.

### 3.2. Microscopic Morphology

**Figure 4:**
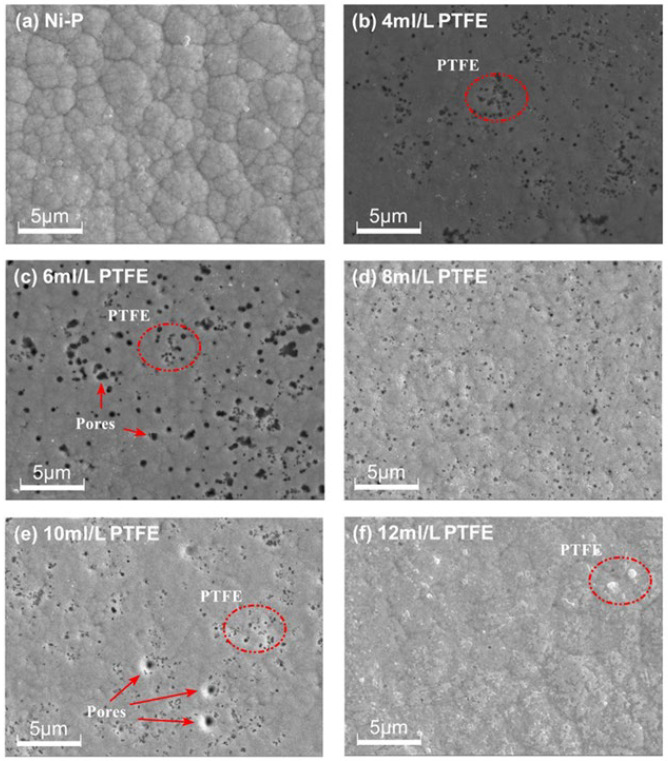
Surface topography of Ni-P coating and Ni-P-NanoPTFE composite coating.

Figure 4 shows scanning electron microscopy images of the surface morphology of Ni-P-nanoPTFE composite coatings prepared under the conditions of the Ni-P coating and with five different PTFE emulsion concentrations (4, 6, 8, 10, 12 mL/L).

It can be seen from Figure 4 that the surface structure of the Ni-P coating is composed of many closely arranged irregular unit cells of different sizes; the boundaries between the unit cells are clear and there are almost no pores on the surface. On the surface of the Ni-P-nanoPTFE coating, there are black PTFE dots and a small number of tiny holes. Compared with the Ni-P coating, the original obvious unit cell structure almost disappears. Comparing the surface morphology of the Ni-P-nanoPTFE composite coatings obtained by five groups of different PTFE emulsion concentrations, it can be seen that the distribution of point-like PTFE particles on the surface of the composite coatings with a PTFE emulsion concentration of 8 mL/L is the most uniform, and there are few pores. The distribution of dotted PTFE particles on the surface of the composite coating at a PTFE emulsion concentration of 12 mL/L is the lowest, and there are sporadic and large white PTFE clusters embedded in the coating. At other PTFE emulsion concentrations, the point-like PTFE particles tend to cluster together, and there are a small number of holes. During the deposition process, the nano-PTFE particles in the plating solution appear to continuously fill the boundary of the single, fine nickel–phosphorus alloy nucleus unit, hindering the mutual merging of the nickel-phosphorus alloy nucleus and resulting in the disappearance of the unit cell structure. Furthermore, during the accumulation process, some nano-PTFE particles co-deposited on the nickel–phosphorus alloy will fall off due to stirring, causing holes in the composite coating.

**Figure 5:**
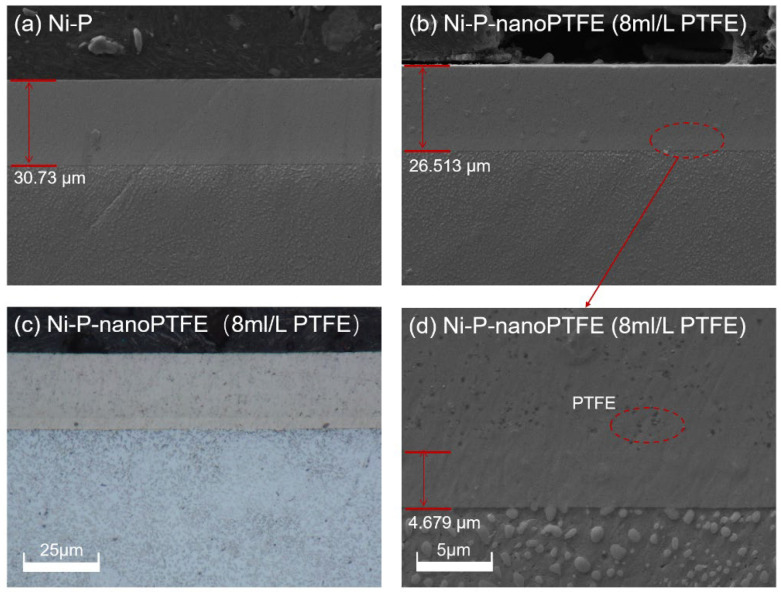
Micromorphology diagram of the cross-section of Ni-P coating and Ni-P-NanoPTFE composite coating under 8 mL/L PTFE concentration.

Figure 5 shows cross-sectional morphology images of a Ni-P coating and a Ni-P-nanoPTFE composite coating prepared at a PTFE emulsion concentration of 8 mL/L, obtained under a scanning electron microscope and a metallographic microscope.

Figure 5 shows that the Ni-P coating and the Ni-P-nanoPTFE composite coating are even and smooth. The Ni-P coating is very tightly combined with the substrate and has a distinct boundary, whereas the boundary between the Ni-P transition layer and the Ni-P-nanoPTFE composite coating is somewhat fuzzy. Using scanning electron microscopy (Figure 5b), it can be observed that the two are vaguely delaminated, whereas under the metallographic microscope (Figure 5c), the two are delaminated. The Ni-P transition layer is white, and the Ni-P-nanoPTFE coating is white with grey. In Figure 5a, the deposition time of the Ni-P coating is 1 h, and the thickness of the coating is 30.73 μm, so the deposition rate of the Ni-P coating is 20.49 μm/h. The total coating thickness in Figure 5b is 26.513 μm, and the Ni-P transition layer thickness in Figure 5d is 4.679 μm. The deposition rate of the Ni-P-nanoPTFE composite coating is 14.556 μm/h, and the introduction of nano-PTFE particles reduces the deposition rate of the original nickel-phosphorus alloy significantly.

### 3.3. Microhardness and Deposition Rate

**Figure 6:**
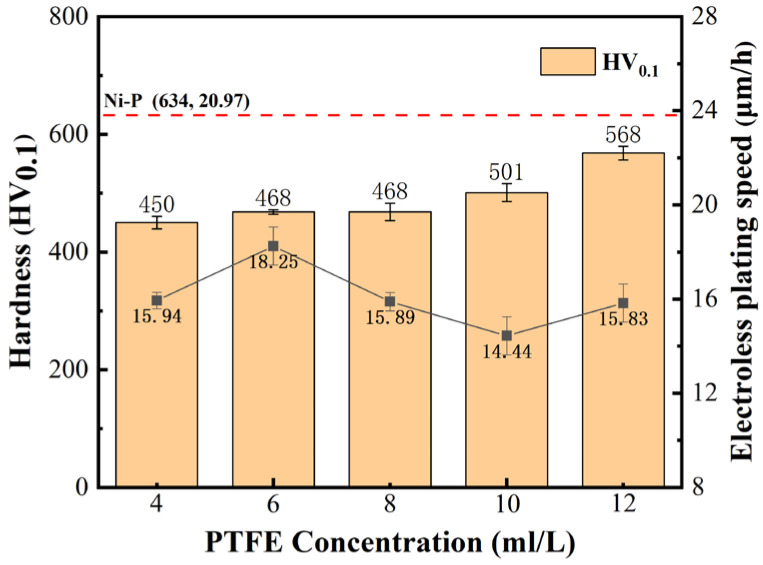
Microhardness histogram and deposition velocity line plot of Ni-P-nanoPTFE coatings prepared at different PTFE emulsion concentrations.

Figure 6 is a histogram of the microhardness and a line graph of the deposition rate of the Ni-P-nanoPTFE composite coating prepared under the five different PTFE emulsion concentrations. The red dotted line in the figure marks the microhardness and deposition rate of the Ni-P coating obtained without the addition of PTFE emulsion, which is 634 HV_0.1_ and 20.97 μm/h, respectively.

It can be seen from Figure 6 that the microhardness and deposition rate of Ni-P-nanoPTFE composite coatings prepared by five groups of PTFE emulsions with different concentrations are lower than those of the original Ni-P coatings. The hardness of the composite coating is approx. 500 HV_0.1_, and the deposition rate fluctuates at approx. 16 μm/h. Within the PTFE emulsion concentration range of 4–12 mL/L, the microhardness of the composite coating increases with the increase in the PTFE emulsion concentration. The deposition rate of the composite coating shows a trend of first increasing, followed by decreasing, and then increasing with the increase in PTFE emulsion concentration. The maximum deposition rate achieved is 18.25 μm/h at a PTFE emulsion concentration of 8 mL/L, and the minimum deposition rate achieved is 14.44 μm/h at a PTFE emulsion concentration of 10 mL/L. Nano-PTFE particles do not participate in the chemical reaction in the plating solution and only rely on weak physical adsorption to stay on the surface of the substrate and co-deposit with the nickel–phosphorus alloy. The molecular groups composed of PTFE particles and surfactants in the plating solution reduce the probability of nickel ions and reducing agents colliding [31], resulting in a significant decrease in the deposition rate of the composite coating. The hardness of the soft nano-PTFE particles uniformly dispersed in the coating is extremely low, which reduces the microhardness of the composite coating [32]. The reason why the deposition rate and microhardness deposition change with the PTFE emulsion concentration is that the relationship between the concentration of surfactant and the dispersion behavior of PTFE particles in the plating solution is not a simple linear relationship. A suitable surfactant concentration can improve the dispersion of PTFE particles: the insufficient active agent will cause part of the PTFE particles to agglomerate, affecting the plating speed, and an excessive active agent will cover the active center on the substrate and hinder the deposition of the coating; however, it should be noted that the use of surfactants should be minimized while maintaining the concentration dispersibility of the current PTFE emulsion.

### 3.4. Phase Structure and Chemical Composition

**Figure 7:**
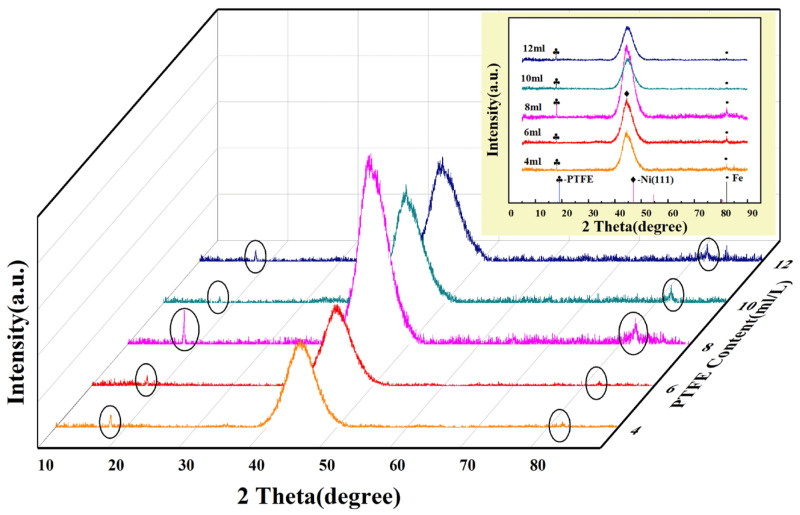
X-ray diffraction spectra of Ni-P-nanoPTFE composite coatings prepared at different PTFE emulsion concentrations. The circle circles the peaks of the two phases of PTFE and Fe.

Figure 7 is the X-ray diffraction spectrum of the Ni-P-nanoPTFE composite coating prepared using the five groups of PTFE emulsion concentrations.

Figure 7 shows that the XRD spectra of the five groups of experiments have essentially the same shape, with sharp peaks appearing near the left side of 2θ = 20°, and steamed-bun-shaped diffuse diffraction peaks appearing near 2θ = 45°. Comparing the two diffraction peaks with the standard card, it is found that they are consistent with the diffraction peak of PTFE at 2θ = 18.08° and the diffraction peak of the Ni (111) crystal plane at 2θ = 44.5°, indicating that PTFE exists in the Ni-P-nanoPTFE composite coating phase and nickel phase. The diffraction peak position of the Ni(111) crystal face is consistent with the XRD spectral results from previous studies [33]. The diffraction peak of 2θ = 85° corresponds to the iron diffraction peak in the matrix material. From the diffraction peak intensities of the five groups of experiments, the two diffraction peak intensities of the composite coating prepared at a PTFE emulsion concentration of 8 mL/L are significantly higher than those of the other four groups. Considering the diffraction peak of nickel, the diffuse reflection peak indicates that the coating structure is amorphous, and the sharp peak represents the crystalline state. The composite coating prepared with a PTFE emulsion concentration of 8 mL/L tends to change from the amorphous state to the crystalline state. Partial crystallites may already exist in the composite coating.

**Figure 8:**
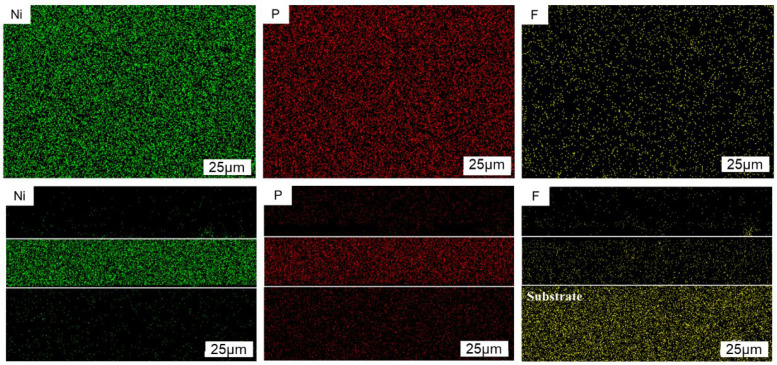
Distribution map of the elements of the Ni-P-nanoPTFE composite coating surface and cross-section, prepared at a PTFE emulsion concentration of 8 mL/L.

Figure 8 is the distribution diagram of Ni, P, and F elements obtained by mapping the surface and cross-section of the Ni-P-nanoPTFE composite coating prepared at a PTFE emulsion concentration of 8 mL/L under the EDS module of the scanning electron microscope. Among them, the Ni element is marked as greenish, the P element is marked as red, and the F element is marked as yellow. The white framed area in the figure is the location of the cross-section of the composite coating.

From the distribution results of Ni and P elements on the surface and cross-section of the composite coating, it can be seen that nickel and phosphorus elements are evenly distributed in the coating. Based on the results of F element distribution on the surface and in the cross-section, it can be seen that the PTFE particles are continuously adsorbed on the surface of the substrate and wrapped by nickel-phosphorus alloy during the entire deposition process, and, finally, evenly dispersed throughout the entire composite coating. From the scanning results of the F element in the cross-section, it can be seen that there is also a large, highlighted area in the matrix part, which proves that there is interference between the F and Fe elements. If the content of PTFE particles in the composite coating is determined, the surface scanning results should be the main ones.

**Figure 9:**
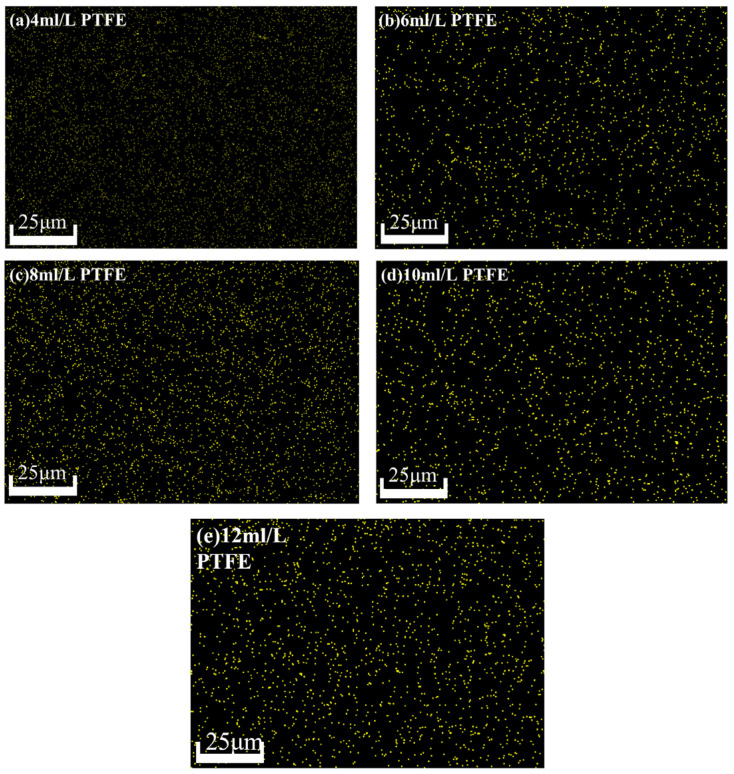
EDS mapping diagrams of surface F element of the composite coating prepared at 4–12 mL/LPTFE emulsion concentration.

Figure 9 shows the distribution of the F element on the surface of the composite coating prepared at five groups of different PTFE emulsion concentrations.

From Figure 9, we can see that in addition to the concentration of 4 mL/L, the F element on the surface of the composite coating prepared under the remaining four groups of concentrations is brighter, more obvious, and contains more content. The distribution of F elements on the surface of the composite coating prepared under five groups of PTFE emulsions with different concentrations was relatively uniform, which proved that the nano-PTFE particles could be uniformly dispersed in the composite coating under the preparation process. Moreover, the F element distribution at the concentration of 8 mL/LPTFE emulsion was the densest and most uniform.

Table 3 shows the Ni, P, and F element mapping results of the composite coatings obtained for the five groups of different PTFE emulsion concentrations.

Considering the results in Table 3, within the PTFE emulsion concentration range of 4–12 mL/L, as the concentration of PTFE emulsion increases, the content of PTFE in the composite coating essentially shows a trend of first increasing and then decreasing. Among them, the Ni-P-nanoPTFE composite coating prepared under a PTFE emulsion concentration of 8 mL/L had the highest PTFE content, up to 2.16 wt%. The main reason for this phenomenon is that when the concentration of the PTFE emulsion is low relative to the surfactant, the excess surfactant is easily adsorbed onto the surface of the substrate. The active centers on the shielding substrate hinder the co-deposition of the nickel–phosphorus alloy and PTFE particles, resulting in a lower content of PTFE in the composite coating. When the PTFE emulsion concentration exceeds that of the surfactant, the surfactant’s dispersion ability cannot meet the requirements of the dispersion of too many PTFE particles in the plating solution. The agglomeration of some PTFE particles hinders the co-deposition of the nickel–phosphorus alloy and PTFE particles, resulting in a low PTFE content in the composite coating.

### 3.5. Wear Properties

**Figure 10:**
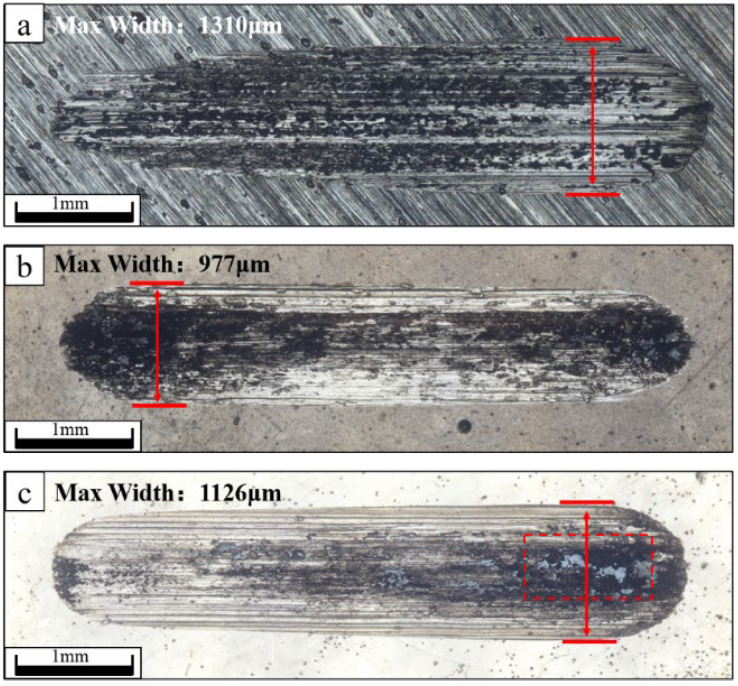
The topography of wear marks of the GCr15 steel substrate, the Ni-P coating, and the Ni-P-nanoPTFE composite coating, prepared with an 8 mL/L PTFE emulsion. The gray substance in the red dotted frame is the grinding chip of the Ni-P-nanoPTFE composite coating.

Figure 10a–c shows the GCr15 steel substrate, the Ni-P coating sample, and the Ni-P-nanoPTFE composite coating sample prepared under a PTFE emulsion concentration of 8 mL/L, respectively.

It can be seen from Figure 10 that the wear scars of the GCr15 substrate are in the shape of a comet, while the wear scars of the Ni-P coating and the Ni-P-nanoPTFE composite coating samples are generally in the shape of long rods with semicircular ends. The long, rod-shaped wear scar conforms to the theoretical wear scar shape of the reciprocating friction test, while the comet-like wear scar is caused by the poor wear resistance of the GCr15 matrix. In the reciprocating friction test, the GCr15 ball of the friction pair penetrated the matrix continuously and rapidly, causing a gradual increase in the resistance it received, finally resulting in the continuous decrease in the reciprocating stroke. Based on the wear debris of the three, the wear debris of the GCr15 matrix is bonded to most of the wear scar area, showing an oxidized black color. The wear debris of the Ni-P coating sample gathers at the two ends of the wear scar, also showing an oxidized black color. The wear debris of the Ni-P-nanoPTFE composite coating remains at one end of the wear scar, showing an off-white color. This indicates that the wear debris of the composite coating has certain self-lubricating properties. After the composite coating is worn, the white nano-PTFE particles in the wear debris are mixed with the oxidized nickel–phosphorus alloy wear debris and appear off-white. Nano-PTFE particles have the characteristics of self-lubrication and a low coefficient of dynamic friction, so the grey-white wear debris acts as a lubricant during the reciprocating friction process and continuously moves with the contact surface. The maximum widths of the three wear scars are 1310, 977, and 1126 μm, respectively, and the smaller the maximum width, the better the wear resistance. From the above perspective, the wear resistance of the Ni-P-nanoPTFE composite coating is slightly lower than that of the Ni-P coating, but better than that of the GCr15 steel substrate, and the nano-PTFE particles in the composite coating can endow the coating with self-lubricating properties.

**Figure 11:**
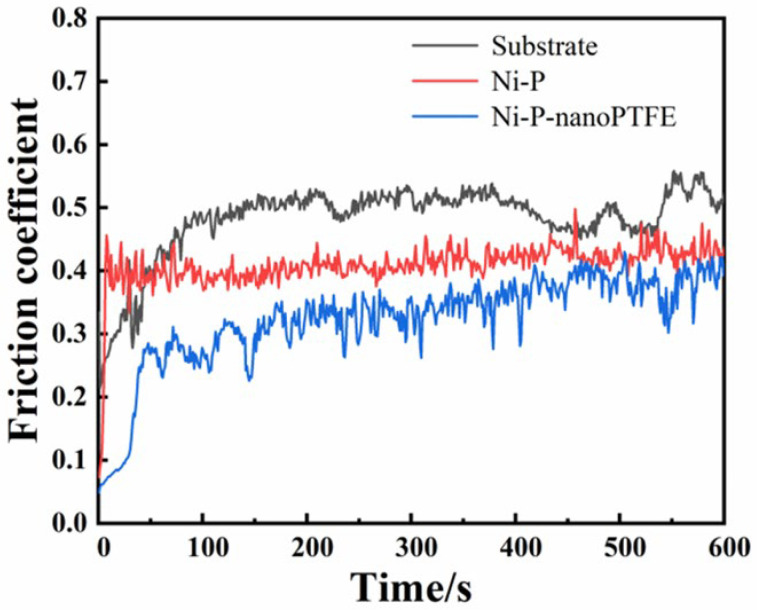
Curves of the friction coefficients of the matrix, Ni-P coating, and NI-P-NanoPTFE composite coating as a function of time.

Figure 11 is a curve diagram of the friction coefficient obtained by the reciprocating friction test of three samples, with a time scale of 10 min.

It can be seen from Figure 11 that the initial friction coefficient value of the GCr15 matrix is approx. 0.2, which then continues to rise and stabilizes at approx. 0.5 in the first 100 s. The initial value of the friction coefficient of the sample coated with Ni-P coating is about 0.08, and then climbs and stabilizes at about 0.4 in a very short time. The initial value of the friction coefficient of the sample coated with Ni-P-nanoPTFE composite coating is approx. 0.07, which then increases to 0.28 in a short time, and then continues to rise slowly to approx. 0.4. The results show that the friction reduction effect of the Ni-P-nanoPTFE coating is better than that of the Ni-P coating.

**Figure 12:**
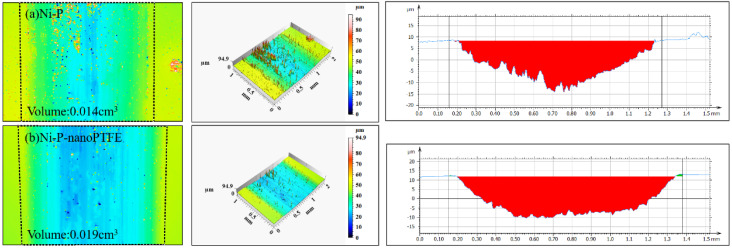
Three-dimensional topography of the wear trace of the Ni-P and Ni-P-NanoPTFE samples.

Figure 12 is the local wear volume, roughness, and cross-sectional shape diagram of the wear scars obtained by the reciprocating friction test of the Ni-P coating sample and the Ni-P-nanoPTFE composite coating sample using laser confocal microscopy.

In Figure 12, it can be observed that the wear volumes of the Ni-P-nanoPTFE composite coating sample and the Ni-P coating sample are 0.019 cm^3^ and 0.014 cm^3^, respectively; the wear volume of the composite coating sample is slightly larger than that of the Ni-P coating. The cross-section of the wear scar of the Ni-P coating sample is in the shape of an inverted triangle, and the bottom of the wear scar is rough and uneven. The cross-section of the wear scar of the composite coating sample is in the shape of an inverted trapezoid, and the bottom of the wear scar is relatively smooth.

In summary, the maximum width of the wear mark of the composite coating is slightly larger than that of the Ni-P coating, and the wear volume is slightly higher than that of the Ni-P coating; however, the wear surface of Ni-P-nanoPTFE composite coating after wear is smoother, and the worn wear chip can improve the lubrication conditions during friction and effectively reduce the friction coefficient, which is beneficial to the application of the coating on the steel collar. When the steel collar and the wire ring are used together, there is a running-in period, during which the fiber-chip lubrication film needs to be quickly formed between the steel collar and the wire ring, and the friction coefficient and smooth running track should be maintained.

### 3.6. Corrosion Properties

**Figure 13:**
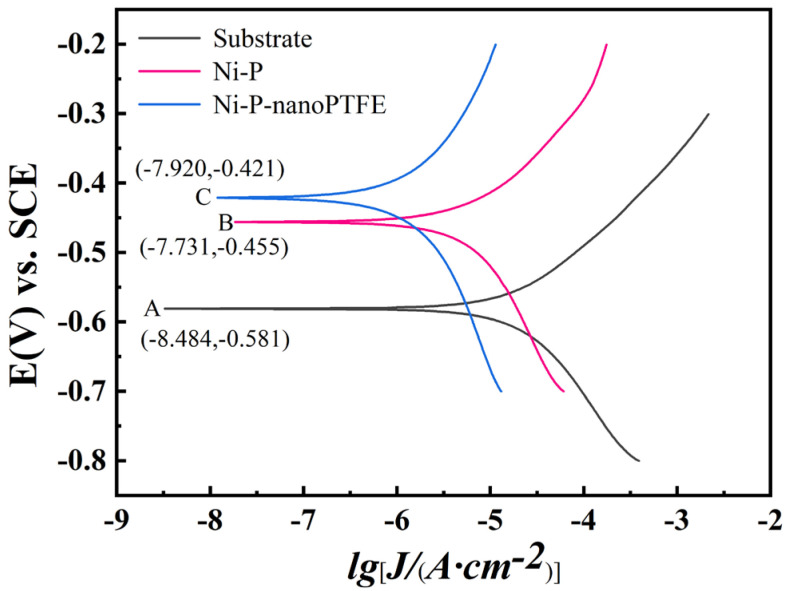
Polarization curves of the GCr15 matrix, the Ni-P coating, and the Ni-P-nanoPTFE composite coating specimens.

Figure 13 is the polarization curve obtained from the electrochemical corrosion test of the GCr15 steel substrate, the Ni-P coating sample, and the Ni-P-nanoPTFE composite coating sample prepared at a PTFE emulsion concentration of 8 mL/L in a 3.5% NaCl solution. Table 4 gives the electrochemical corrosion parameters after fitting the three polarization curves.

Table 4 shows that the absolute values of the anodic slopes of the three samples’ polarization curves are greater than the absolute values of the cathodic slopes, indicating that all three function using the anodic protection mechanism. The corrosion potential of the Ni-P coating and the Ni-P-nanoPTFE composite coating samples shifted toward a more positive value compared with the GCr15 steel substrate. In addition, the corrosion current density gradually decreases, and the polarization resistance gradually increases, compared with the GCr15 steel substrate. Note that in Figure 13, the GCr15 substrate, the Ni-P coating sample, and the Ni-P-nanoPTFE composite coating sample correspond to A, B, and C, respectively. The order of the corrosion potential, the corrosion current density, and the polarization resistance of the three are E_A_ < E_B_ < E_C_; I_A_ > I_B_ > I_C_; and R_A_ < R_B_ < R_C_, respectively. Among them, after the GCr15 substrate was coated with the Ni-P-nanoPTFE composite coating, the corrosion current density decreased from 19.70 μA to 1.54 μA, the polarization resistance increased from 1460 Ω to 27,074 Ω, and the difference between the two indicators was one order of magnitude. The higher the corrosion potential, the lower the possibility of corrosion; the lower the corrosion current density, the slower the corrosion rate; and the higher the polarization resistance, the better the corrosion resistance [34]. Considering the polarization curve, the corrosion resistance of the Ni-P-nanoPTFE composite coating is not only better than that of the Ni-P coating but also significantly better than that of the GCr15 substrate.

**Figure 14:**
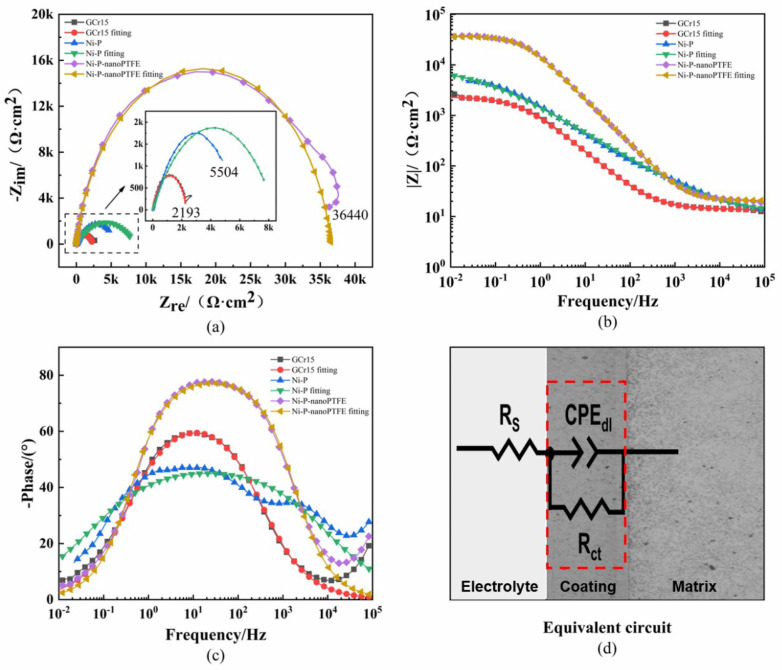
Nyquist and Bode diagrams of three specimens and the equivalent circuit diagram. (**a**) is the Nyquist diagram, (**b**) is the bode diagram, (**c**) is the phase diagram, and (**d**) is the equivalent circuit diagram.

Figure 14 shows the Nyquist and Bode diagrams and the fitted equivalent circuit diagrams obtained from electrochemical corrosion tests of three samples in a 3.5% NaCl solution. Table 5 gives the fitting results of the component parameters of the equivalent circuit diagram.

Figure 14 and Table 5 indicate that the fitting results of the GCr15 substrate and the Ni-P-nanoPTFE composite coating sample are better than those of the Ni-P coating sample, which shows a slight deviation that is mainly reflected in the high-frequency part of the impedance spectrum and phase diagram. The impedance spectra of GCr15 steel, the Ni-P coating sample, and the Ni-P-nanoPTFE composite coating sample are semicircular, with radii of 2193, 5504, and 36,440 Ω·cm^2^, respectively. The radius of the Ni-P-nanoPTFE composite coating sample is an order of magnitude higher than those of the other samples, and the larger the radius, the better the corrosion resistance. It can be seen from the Bode diagram that the impedance modulus of the Ni-P-nanoPTFE composite coating at 0.01 Hz is higher than 10^4^ Ω·cm^2^ and that the impedance moduli of the Ni-P coating sample and the GCr15 substrate at 0.01 Hz are lower than 10^4^ Ω·cm^2^. The larger the impedance modulus in the low-frequency region, the better the corrosion resistance. In the equivalent circuit diagram, R stands for resistance, and CPE stands for the electric double-layer capacitance constant phase angle element, which is characterized by the admittance coefficient Y_0_ and the dispersion index n. Rs is the solution resistance, and CPE_dl_ and R_ct_ are the capacitance and resistance, respectively, caused by the oxide film or the coating on the substrate. The capacitance Y_0_ value of the Ni-P-nanoPTFE composite coating is smaller than that of the substrate, and the resistance R_ct_ value is larger than that of the substrate. The smaller the capacitance value Y_0_, the faster the capacitor is charged, the faster the current channel is disconnected, and the better the corrosion resistance [35]. In general, the corrosion resistance of the Ni-P-nanoPTFE coating is the best, the main reason being that the P element in the nickel–phosphorus alloy coating can undergo an anodic oxidation reaction in a corrosive medium to form a phosphating passivation film. The uniformly distributed nano-PTFE particles in the composite coating are inert and hydrophobic, which can further hinder the corrosion of the substrate by the corrosive medium.

## 4. Conclusions

In summary, during the preparation of Ni-P-nanoPTFE composite coatings, adding KR-XP96 defoamers to the plating solution can effectively inhibit the foaming of surfactants and prevent PTFE particle agglomeration and floating caused by excessive foaming. Pre-depositing a Ni-P transition layer on the surface of the substrate can reduce the possibility of missing plating on the edge. Compared with the Ni-P coating, the deposition rate and microhardness of the Ni-P-nanoPTFE composite coating prepared by this process decreased from 20.97 μm/h and 634 HV_0.1_ to approx. 16 μm/h and 500 HV_0.1_, respectively. This phenomenon is consistent with the previous research results [36], soft PTFE particles will reduce the hardness of Ni-P-nanoPTFE composite coating. The PTFE content in the composite coating prepared at a PTFE emulsion concentration of 8 mL/L was the highest, reaching 2.16 wt%. Compared with Ni-P coating, although the introduction of nano-PTFE has slightly increased the wear volume of the composite coating from 0.014 cm^3^ to 0.019 cm^3^, and the maximum wear width has increased from 977 to 1126 μm, the friction coefficient reduced from approx. 0.4 to approx. 0.3 compared with the Ni-P coating. The PTFE particles mixed with wear debris in the composite coating have self-lubricating properties, and the wear resistance of the Ni-P-nanoPTFE composite coating is better than that of the Ni-P coating. This self-lubricating performance is mainly because nano-PTFE particles are doped into the abrasive chip due to wear. PTFE molecular structure contains many [CF2]-chemical repeating units, low sliding friction coefficient, strong adhesion ability, but has low bonding ability between layers and can bond to the two surfaces of a pair of friction pairs to form two thin layers of lubricating film, thereby reducing friction [30,37], which is consistent with the friction test results of the composite coating in this paper. Abrasive chips containing PTFE lubricate the contact surface between the composite coating and the friction parts. Compared with the Ni-P coating, the corrosion potential of the Ni-P-nanoPTFE composite coating shifted toward a more positive value, from −456 mV to −421 mV, its corrosion current decreased from 6.71 μA to 1.54 μA, and the impedance greatly improved, from 5504 Ω·cm^2^ to 36,440 Ω·cm^2^. The corrosion resistance of the Ni-P-nanoPTFE composite coating is better than that of the Ni-P coating.

The Ni-P-nanoPTFE composite coating in this study is applied to textile steel collars, which can accelerate the formation of abrasive-fiber lubricating film between steel collars and steel wire rings, reduce the friction coefficient between the two, and ensure the smooth operation of steel wire rings during the use stage. In future research work, Ni-P-nanoPTFE composite coatings will be used as sacrificial coatings to be combined with other super hard coatings. This allows for shorter lubrication formation times while maintaining higher wear resistance.

## Figures and Tables

**Table 1:** GCr15 steel chemical composition table (wt%).

C	Cr	Mn	Si	Ni	Cu	P	S	O	Fe
0.95	1.44	0.36	0.27	0.06	0.07	0.13	0.004	0.0004	Bal.

**Table 2:** Ni-P-nanoPTFE electroless plating bath composition and process parameters.

Chemical Components or Technological Condition	Concentration or Parameter	
	Ni-P Coating	Ni-P-PTFE Coating
NiSO_4_·6H_2_O	25 g/L	25 g/L
NaH_2_PO_2_·H_2_O	29.2 g/L	29.2 g/L
C_6_H_8_O_7_·H_2_O	5 g/L	5 g/L
C_3_H_6_O_3_	25 mL/L	25 mL/L
NaC_2_H_3_O_2_·3H_2_O	15 g/L	15 g/L
H_2_NCSNH_2_	1 ppm	1 ppm
CTAB	-	30 ppm
FC-4430	-	90 ppm
KR-XP96	-	60 ppm
PH	4.4	4.4
Temperature	85 ± 2 °C	85 ± 2 °C
Time	10 min, 1 h	90 min

**Table 3:** Surface element distribution diagram of Ni-P-nanoPTFE composite coating prepared with different PTFE concentrations (wt%).

	PTFE	4 mL/L	6 mL/L	8 mL/L	10 mL/L	12 mL/L
Element						
F		1.66	1.78	2.16	1.27	1.40
P		9.41	8.58	9.81	8.47	7.12
Ni		88.60	89.44	87.82	90.08	90.19
Fe		Balance				

**Table 4:** Electrochemical corrosion parameters after polarization curve fitting.

Sample	−E_corr_/mV	lg [J_corr_/(μA·cm^−2^)]	R_p_/Ω	−b_c_/(mV·dec^−1^)	b_a_/(mV·dec^−1^)
Substrate	581	19.70	1460	6.06	9.05
Ni-P	456	6.71	5302	4.83	7.39
Ni-P-nanoPTFE	421	1.54	27,074	4.86	5.61

Note: E_corr_ is corrosion potential, J_corr_ is corrosion current density, b_c_ is cathode Tafel slope, and b_a_ is anode Tafel slope.

**Table 5:** Equivalent circuit fitting parameters.

Sample	R_s_/(Ω·cm^2^)	Y_0_/(Ω^−1^·cm^−2^·S^−n^)	n	R_Ct_/(kΩ·cm^2^)
Substrate	13.74	2.34×10^−4^	0.7482	2.339
Ni-P	11.78	2.35×10^−4^	0.5347	8.380
Ni-P-nanoPTFE	20.76	1.20×10^−5^	0.8867	36.532

## Data Availability

Not applicable.
